# Microarray-based identification and RT-PCR test screening for epithelial-specific mRNAs in peripheral blood of patients with colon cancer

**DOI:** 10.1186/1471-2407-6-250

**Published:** 2006-10-20

**Authors:** Rossella Solmi, Giampaolo Ugolini, Giancarlo Rosati, Simone Zanotti, Mattia Lauriola, Isacco Montroni, Marco del Governatore, Antonello Caira, Mario Taffurelli, Donatella Santini, Domenico Coppola, Lia Guidotti, Paolo Carinci, Pierluigi Strippoli

**Affiliations:** 1 Department of Histology, Embryology and Applied Biology, University of Bologna, Via Belmeloro 8, I-40126 Bologna, Italy; 2 Department of Surgical and Anesthesiological Sciences-General Surgery, University of Bologna, Via Massarenti 9, I-40138 Bologna, Italy; 3 "H.Lee Moffit" Cancer Center and Research Institute, University of South Florida, Tampa, FL, USA; 4 Department of Pathology, University of Bologna, Via Massarenti 9, I-40138 Bologna, Italy

## Abstract

**Background:**

The efficacy of screening for colorectal cancer using a simple blood-based assay for the detection of tumor cells disseminated in the circulation at an early stage of the disease is gaining positive feedback from several lines of research. This method seems able to reduce colorectal cancer mortality and may replace colonoscopy as the most effective means of detecting colonic lesions.

**Methods:**

In this work, we present a new microarray-based high-throughput screening method to identifying candidate marker mRNAs for the early detection of epithelial cells diluted in peripheral blood cells. This method includes 1. direct comparison of different samples of colonic mucosa and of blood cells to identify consistent epithelial-specific mRNAs from among 20,000 cDNA assayed by microarray slides; 2. identification of candidate marker mRNAs by data analysis, which allowed selection of only 10 putative differentially expressed genes; 3. Selection of some of the most suitable mRNAs (*TMEM69*, *RANBP3 *and *PRSS22*) that were assayed in blood samples from normal subjects and patients with colon cancer as possible markers for the presence of epithelial cells in the blood, using reverse transcription – polymerase chain reaction (RT-PCR).

**Results:**

Our present results seem to provide an indication, for the first time obtained by genome-scale screening, that a suitable and consistent colon epithelium mRNA marker may be difficult to identify.

**Conclusion:**

The design of new approaches to identify such markers is warranted.

## Background

Early detection seems to be a key factor in reducing rates of death from colorectal cancer [1], one of the commonest cancers in the world [2]. Current methods of screening include fecal occult blood testing (FOBT), flexible sigmoidoscopy, barium enema, and colonoscopy. Recently, "virtual" (computed tomographic) colonography has been proposed as a relatively non-invasive alternative to colonoscopy for detecting colorectal neoplasia [3]. In addition, novel methods of detecting common molecular alterations in colorectal cancer cells, such as methylation changes in fecal DNA [4], are being evaluated. However, none of these methods is currently widely used for screening the general population, due either to patient discomfort or low sensitivity/specificity.

The search for epithelial cells as a screening tool in the patient's blood represents an important field of research for early detection of epithelial cancers. The rationale for using this as a colorectal cancer screening method lies in the fact that solid tumors as small as 2 mm diameter typically display active angiogenesis [5] and hence are capable of releasing tumor cells into the peripheral blood; in the earlier stages of the disease, disseminated cells are not capable of forming metastases, but they may provide a clue for cancer detection [6].

The concept of circulating tumor cell (CTC) detection has so far been put forward for breast cancer in particular. Several authors in this field have established that: CTCs are rare events occurring at a frequency of approximately one tumor cell per 1× 10^5–7 ^peripheral blood mononuclear cells [7]; methods to identify CTCs must distinguish between epithelial and hematopoietic cells in blood while it may not be essential to distinguish between cancer and normal epithelial cells [8]; selection based upon physical properties such as morphology, size, and weight have limitations in both sensitivity and specificity [8]. A new system proposed for breast cancer is based on counting epithelial cells, which are separated from the blood by antibody-coated magnetic beads and identified using fluorescent labeled antibodies against cytokeratin, as well as a fluorescent nuclear stain and fluorescent cytokeratin antibodies [9].

Detection of cancer cells in the blood could employ an epithelial-specific mRNA, which might be revealed in the patient's blood sample via amplification by RT-PCR (reverse transcription-polymerase chain reaction). This approach has been applied starting both from unfractioned whole blood [10-13] and from blood fractions, e.g. separated mononuclear cells or immunomagnetically enriched cancer cells [14-22].

While using RT-PCR could overcome the problems of lack of sensitivity associated with other methods of identification, the selection of epithelial-specific mRNA is difficult. Previous studies on this topic have mostly been performed in relation to breast cancer, and only a few studies have included colorectal cancer [12,15,20-28]. A recent review [29] has led to the conclusion that methods of identifying epithelial specific mRNA markers are not reliable at this point, and need additional study.

In a previous work describing a bioinformatic method aimed at identifying putative epithelial-specific mRNAs suitable for detection of colorectal CTC in the blood, we showed that for all the 15 genes investigated it failed to distinguish between normal and patients' blood by qualitative RT-PCR [30].

In this work, we present a new microarray-based high-throughput screening approach to identifying candidate marker mRNAs for early detection of epithelial cells diluted in peripheral blood cells. This method included: direct comparison of different samples of colon mucosa and blood cells, searching for epithelial-specific genes among the 20,000 genes assayed by microarray slides; identification of candidate marker mRNAs by data analysis, which allowed for only 10 putative differentially expressed genes; selection of some of the most suitable mRNAs (*TMEM69*, *RANBP3 *and *PRSS22*) that were assayed in blood samples from normal subjects and patients with colon cancer as possible markers for the presence of epithelial cells in the blood, using RT-PCR. None of the 3 candidate mRNAs was proven to be absent from the blood of normal subjects. This first study based on a systematic search for epithelial-specific mRNAs in the blood reveals how difficult it is to identify robust indicators of tissue-specific expression.

## Methods

### RNA sources

A peripheral blood sample (10–15 mL) was obtained from 2 patients on the brink of surgery and from 4 healthy donors (Table 1). The study was conducted in compliance with the Helsinki Declaration, it was approved by the senior staff committee, a board regulating non-interventional study comparable to an institutional review board; all patients and subjects involved gave their written informed consent. To reduce contamination of samples with skin epithelial cells from the needle stick, the first mL of blood was discarded. Then 10–15 mL of whole blood, drawn into an EDTA tube, was treated for RNA extraction within one hour of being drawn, as described below. We also used total RNA samples from: a) normal human peripheral leukocytes pooled from 250 male/female Caucasians, aged 18–40 (BD-Biosciences, Clontech, Palo Alto, CA); b) normal human colon with mucosal lining pooled from a suddenly deceased 35 year old Caucasian female (BD-Biosciences); c) colon adenocarcinoma isolated from a 28 year old Caucasian male) (BD-Biosciences); d) normal human colon and adenocarcinoma isolated from an 82 year-old male (mucosa margin respectively disease free and adenocarcinoma-bearing, Stratagene, La Jolla, CA); e) normal human colon pooled from three males aged 50, 76 and 94 (Stratagene, La Jolla, CA).

### Automated RNA extraction

Total cellular RNA was extracted from peripheral blood samples using the ABI PRISM 6100 system. This new 96-well platform instrument is engineered for run-to-run and well-to-well high-yield purification that is reproducible and free of cross-contamination. Cellular lysates or homogenized tissue lysates are added to the tray and vacuum is applied under user interface control for a specific time and at an electronically controled pressure.

Blood samples were lysed within one hour of being drawn, diluting 1 mL of whole blood with 1 mL of phosphate buffered saline (PBS) and adding 2 mL of lysis solution provided by the manufacturer. The lysate was stored at -20°C up to RNA extraction by ABI PRISM 6100. Extraction was performed according the manufacturers' instructions. Total RNA extracted from 1 mL of peripheral blood was subjected to standard ethanol precipitation, and the pellet was re-suspended in 20 μL of steril bidistillated water and stored at -20°C.

### DNA microarray screening and analysis

Comparison of blood and colon global gene expression was performed using hybridization of the microarray MWG Pan Human 40K array A, an oligonucleotide-based array on a glass slide containing 20,000 gene-specific probes (50 mer) (MWG Biotech AG, Ebersberg, Germany).

Gene expression analysis was performed by a process of RNA extraction, reverse transcription and labeling of cDNA. Reference (i.e. blood-derived) and investigated (i.e. colon mucosa-derived) cDNA are in this process labeled with different dyes and then co-hybridized on slides containing cDNA fragments. The slides are then scanned with a laser system, and two false color images are generated for each hybridization with cDNA from the investigated and reference sample. The overall result is the generation of a so-called genetic portrait.

RNA was purchased or extracted as described above. Ten micrograms of total RNA were used for each sample. cDNA was synthesized using a CyScribe Post-Labeling Kit Cat. no RPN5660 (Amersham Biosciences, Europe, Freiburg, Germany) that provides reagents for preparation of Cy3 and Cy5 labeled cDNA probes. The first step involves the incorporation of aminoallyl-dUTP (AA -dUTP) during cDNA synthesis using an optimized nucleotide mix, RNA hydrolysis and purification of amino allyl-modified cDNA, while a second step involves chemically labeling the amino allyl-modified cDNA using the CyDye NHS-esters provided and purifying CyDye-labelled cDNA with autoseq G-50 columns (CyScribe GFX Purification kit Cat no.27-9606-02, Amersham Biosciences, Europe, Freiburg, Germany).

We added respectively 130 μL of preheated hybridization buffer to Cy3- or Cy5-fluorescently labeled reference, and proceeded to investigate cDNA. For hybridization we used the microarray "Gene Frame" as a cover slip to prevent reagent loss due to evaporation during incubation at 37°C overnight.

In our three independent hybridization experiments: i) normal human peripheral leukocytes pooled from 250 male/female Caucasians cDNA were labeled with Cy5 and normal human colon with mucosal lining pooled from a 35-year old Caucasian female cDNA was labeled with Cy3; ii) locally extracted human peripheral blood RNA pooled from 4 normal Caucasians (Table 1, subjects no. 3, 4, 5 and 6) cDNA was labeled with Cy5 and colon adenocarcinoma isolated from a 28-year old male Caucasian cDNA was labeled with Cy3; iii) locally extracted human peripheral blood pooled from 2 patients with colon cancer cDNA was labeled with Cy3 and cDNA from adenocarcinoma disease isolated from an 82-year old male was labeled with Cy5. We later refer to these experiments as slide nos. 1, 2 and 3, respectively.

Washing was performed three times for 10 min with 1× saline sodium citrate (SSC), 0.1% sodium dodecyl sulfate (SDS) at 42°C, and three times for 5 min with 0.1× SSC at room temperature. Slides were dried by centrifugation for 2 min at 2000 rpm.

### Array image and data analysis

A GenePix 4000a DNA microarray scanner (Axon, Union City, CA, USA) was used to scan the slides under dried conditions. The laser power for scanning green and red colors was adjusted in order to obtain a global intensity ratio near to 1. If necessary, further washes were performed to reduce the aspecific background.

Each spot was defined using the grid schema provided by the manufacturer, with manual adjustment for the positioning of spot blocks. Spots showing no signal or obvious defects were accordingly flagged by visual inspection and excluded from analysis.

Data were extracted with GenePix Pro3.0 software (Axon Instruments). The median of the pixel intensities for each spot and for the corresponding surrounding area (background) was obtained for each channel.

We imported data into the FileMaker Pro 8 database (FileMaker Inc., Santa Clara, CA) so as to search for genes consistently over-expressed in colon versus blood tissue in all three experiments performed.

### Reverse transcription-polymerase chain reaction (RT-PCR) amplification

Total RNA (about 2.25 μg) from peripheral blood or from human colon was reverse transcribed in a final volume of 50 μL in the presence of: 500 mM each of deoxynucleotide triphosphate (dNTP), 200 U reverse transcriptase (Murine Moloney Leukemia Virus) with companion buffer 1 × (Promega, Madison, WI, U.S.A.), oligo dT-15 5 μM. The incubation was performed at 42°C for 1 h, followed by denaturation at 95°C for 5 min.

The primers for amplification were designed using the software Amplify [31], following standard criteria. Primer sequences and predicted product size are given in Table 2.

PCR experiments were performed in 25 μL final volume, containing 3 μL RT mix, 1U Taq Polymerase (TaKaRa, Shiga, Japan) with companion reagents (0.2 mM each dNTP, 1.5 mM MgCl_2_, 1× PCR buffer), and 0.3 μM of each primer. An initial denaturation step of 2 min at 94°C was followed by amplification for 30–45 cycles (30 sec at 94°C, 30 sec at 64°C, 30 sec at 72°C) and final extension for 7 min at 72°C. All the RT-PCR products obtained were gel analysed following standard methods [32]: ethidium bromide incorporation in the gel (0.5 μg/mL final concentration), transillumination at 300 nm, and photography.

## Results

### Automated RNA extraction

The automated RNA extraction applied method allowed for quick extraction of a reproducible amount of total RNA from all the samples analysed, confirming our previous results [30]. The amount of total RNA obtained from 1 mL of each peripheral blood sample was about 5 μg, at a concentration of 20 ng/mL. Since the number of circulating tumor cells might be very low, if compared to blood cells, we performed a precipitation step to obtain a final RNA concentration of 0.25 μg/μL. Quality control on agarose minigel confirmed the integrity of total RNA.

### Array image and data analysis

The dataset used as the base for analysis was the "results" file provided by the software GenePix 3. The "background median" was the median of the pixel intensities in the area surrounding the spot, while the "feature median" was the median of the pixel intensities in the area inside the spot. The spot intensity was then calculated by subtracting the background median value from the feature median value. The original datasets for experiments 1, 2 and 3 as defined above are provided as supplementary data [33].

After data importation into a relational database management system (FileMaker Pro 7 for Macintosh), we checked and normalized the data. We used the "total intensity" normalization factor [34] to balance the green/red series of measurements for each slide.

For the analysis, we considered only those genes (out of 20,160 total probes) meeting the following criteria in all three experiments performed: presence of colon-related "intensity" values greater than 0; absence of any related spot visually localized in areas with artifacts, manually flagged by the operator as "bad" (code -100); absence of any related spot flagged as "not found" (code -50) by the GenePix software, by internal criteria including number of pixels above background threshold or feature diameter – usually this means that the expression level cannot be affordably assessed for technical (artifactual area) reasons.

To identify colon-expressed genes that were significantly under-expressed in blood cells, we searched the normalized dataset for all genes whose expression ratio colon/blood was above the best threshold able to identify a criterion-matching gene.

In order to have meaningful numbers when dividing "colon-related" values by "blood-related" values, we made a transformation so that all "blood-related" values equal to or lower than "0" were taken as equal to "1". This enabled values at the lowest range of detection, e.g. "2" in the colon and "1" in the blood, to be measured as at least 2-fold increases. Only a colon value of 1 on a blood value of 0 ratio could go undetected, being transformed into 1/1, but such a ratio would have no significance for our purposes. The normalized and transformed values for the 11,529 genes meeting the criteria above described are tabulated in a file available on line [33].

Searching for the greatest colon/blood ratios in all the experiments, we were able to identify 10 genes matching the above described criteria, which proved to have a ratio of at least 2.8: [GenBank : AF19551 (*PURG*), NM_016486 (*TMEM69*), NM_003624 (*RANBP3*), AB010779 (*PRSS22*), AF080227 (*EED*), BC009048 (*TINAGL1*), BC021574 (*G6PC3*), BC023542 (*SORT1*), BC028610 (*LRRC34*) and M63967 (*ALDH1B1*)].

Useful candidates might have been lost by our analysis when the whole series of three measurements was not available due to slide artifacts. To test this hypothesis, we finally searched for colon mRNAs over-expressed in at least two slides, when the third measurement proved to have been labeled as "bad" by the software (104 and 7,469 spots out of 20,160 in slide nos. 2 and 3, respectively) or by visual inspection (43, 1,533 and 338 spots out of 20,160 in slides 1, 2 and 3, respectively). In this way, only a few additional potential candidates were identified: 4 considering slides no. 2 and 3, 6 for slides no. 1 and 3, and none for slides no. 2 and 3. However, in these cases one or more of the following conditions recurred: values were in the low range of detection for both channels, blood channel values were positive, or colon channel values were "0", thus implying that the low number of mRNA candidates found by analyzing only the values detectable in all three experiments was not due to technical artifacts.

### Reverse transcription-polymerase chain reaction (RT-PCR) amplification

Although we did not find any mRNA with an ideal expression level difference between colon and blood tissues, we chose three genes out of the 10 identified as above to formally exclude their suitability as epithelial markers in the blood. We chose the two genes with the greatest mean colon/blood ratio throughout the three experiments (*RANBP3 and PRSS22*), and we also selected the gene with the greatest absolute expression value in the colon (*TMEM69*). A data summary for these genes is summarized in Table 3.

RT-PCR products of the expected size were all obtained from at least some RNA control subject samples for these three genes.

Gene expression was tested for each gene on total RNA derived from: 4 normal peripheral blood samples (subjects no. 3, 4, 5 and 6 in Table 1, respectively), 1 commercial pool of RNA leukocytes from 250 normal subjects, 1 pool of two colon cancer patients' peripheral blood samples (subjects no. 1 and 2 in Table 1, respectively). Normal and neoplastic colon mucosa were used as controls for gene expression.

**Figure 1:**
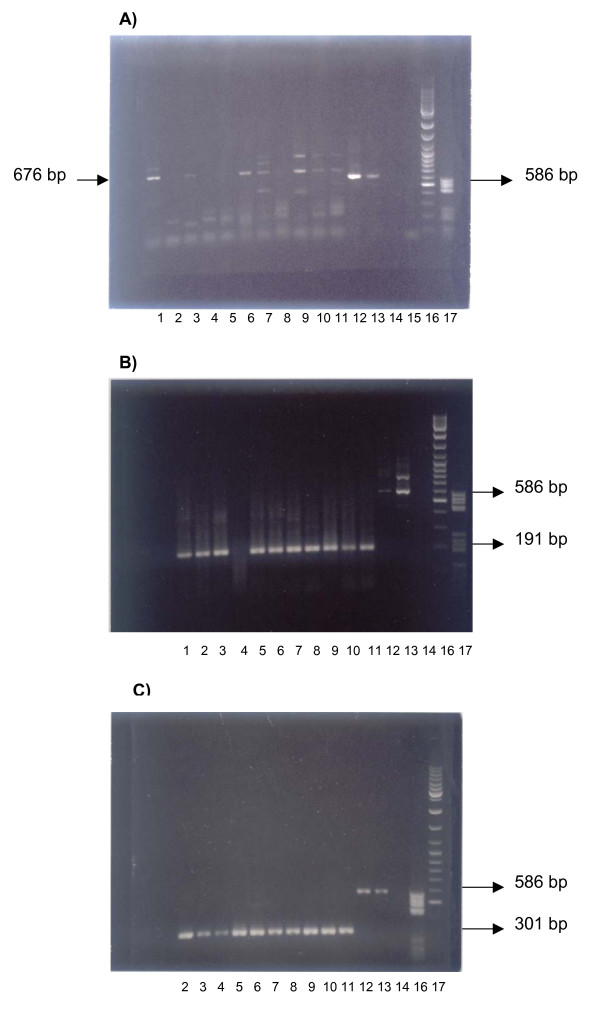
**Ethidium-bromide stained 1% agarose gel of RT-PCR amplification products**. **A)**, *PRSS22*, **B) ***RANBP3 *and **C) ***TMEM69 *gene expression. Lanes for each gene are labeled with the scheme: 1–4, blood RNA of normal individuals (Table 1, subjects no. 3–6, respectively): lane 5, normal leukocytes RNA pooled from 250 subjects (source: BD); lane 6, pooled blood of colon cancer patients (Table 1, patients no. 1–2); lane 7 normal human colon with mucosal lining pooled from a control subject (source: BD); lane 8 colon adenocarcinoma isolated from a colon cancer patient (source: BD); lane 9, pool of normal mucosa from 3 control subjects (source: Stratagene); lanes 10–11, disease-free margin mucosa and colon cancer samples, respectively, from the same patient (source: Stratagene). Technical controls: lanes 12–13, PCR positive control (*B2M *RT-PCR amplification products from the same samples tested in lanes 5 and 9, respectively), lane 14–15 PCR negative (water) control; lanes 16–17, 1 μL molecular size markers MBI V and GeneRuler (Genenco), respectively.

[GenBank : AB010779 (*PRSS22*)] mRNA was detected in 2 out of 5 normal peripheral blood samples (Fig. 1A, lanes 1–5); aspecific amplification was also observed. [GenBank : NM_003624 (*RANBP3*)] mRNA was detected in 4 out of 5 normal peripheral blood samples (Fig. 1B, lanes 1–5). [GenBank : NM_016486 (*TMEM69*)] mRNA was detected in 4 out of 4 tested normal peripheral blood samples (Fig. 1C, lanes 1–4). All genes investigated were expressed in both normal and neoplastic colon mucosa.

## Discussion and conclusion

If tumor cell shedding is an early event in tumorigenesis, it may be possible to detect cancer cells in the bloodstream before the primary tumor is large enough to be detected by standard screening examinations [35]. Although detection of circulating tumor cells (CTCs) in peripheral blood has been of interest for over a century [36], only in recent times has the development of methods to detect them received attention.

The aim of this work was to use gene expression profiling of normal or cancer colon mucosa in comparison with normal or colon cancer patients' peripheral blood, in order to identify a set of genes that could be used for detection of CTCs in the peripheral blood from patients with colorectal cancer, using a standardized PCR test according to the World Health Organization criteria for a screening test: acceptability, practicability, high specificity and high sensitivity [37].

DNA microarray technology allows one to investigate gene expression in parallel for a very large number of genes, and it has the ability to detect a change of expression between two compared cellular samples. In particular, we chose an MWG Pan Human 40K array A (MWG Biotech AG Ebersberg, Germany), a recent type of oligonucleotide array on glass slides containing 20,000 gene-specific oligonucleotide probes. These are particularly suitable for marker selection owing to the big number of known genes spotted on. In three independent hybridization experiments we compared RNA samples deriving from colon cells with RNA samples deriving from peripheral blood cells.

Various popular current approaches try to identify markers of cancer progression by comparing normal and neoplastic colon mucosa. Comparison of global gene expression profiles of purified CTC and CTC-depleted blood from patients with colon cancer has also recently been reported [21], no single mRNA proving to be suitable for identification of CTCs. Our approach exploits a more "biological" concept, focusing on the identification of consistent "epithelial" mRNA markers, which ought not to be present in normal blood.

To this end, we used various different types of comparisons regarding the cancerous/normal state, provided that in each experiment a colon cell sample was compared with a blood cell sample. In addition, we used both single and pooled samples, to favor the detection of consistently up-regulated genes [38,39].

Our analysis failed to identify any single gene that is consistently and strongly over-expressed in colon cells as compared to blood cells. This finding is in agreement with recent data on global gene expression, which point out that a small number of genes may be responsible for even great differences observed in differentiated cells from different tissues. However, we were able to identify the best 10 genes, among 20,000 tested, which gave the best attainable colon/blood expression ratio (at least 2.8:1) in all three experiments. By a simple qualitative RT-PCR assay we tested three of these genes (*TMEM69*, *RANBP3 *and *PRSS22*, suitable for mRNA-specific amplification, being composed of more than one exon) on RNA samples from pooled and individual peripheral blood from control subjects. Interestingly, it has been previously demonstrated that prosemin, the product encoded by *PRSS22 *gene, is expressed and secreted by various kinds of cancer cells, such as glioma, pancreas, prostate, and ovarian cell lines, and the potential usefulness of this serine protease as a candidate tumor marker has been put forward [40].

We confirm the utility of a semi-automated method of RNA extraction (nucleic acid preparation by the ABI PRISM 6100 system, followed by a precipitation step) which allowed us to obtain a standard amount of total RNA from 1 mL of each peripheral blood sample, suitable for RT-PCR [30]. This method is readily amenable to the analysis of 48 samples/run, it employs no toxic reagents and is very fast (30–60 minutes from sample to concentrated, intact total RNA). The positivity of the investigated RNA markers in a small set of blood control samples did not allow us to consider these candidate genes epithelial-specific, excluding them from any role in the identification of cancer cells in patient blood. This finding is not unexpected, due to the absence of genes with an ideal expression level difference, as well as to the need for using as best potential markers genes with a difference of only ~3-fold in a highly sensitive test such as RT-PCR.

In order to compare our finding with previous results concerning "epithelial" mRNA markers, we searched for cytokeratin 20 (*KRT20*) mRNA spot in our array platform, because it has been considered an epithelial marker suitable for the identification of epithelial cells in the blood [41].

The *KRT20 *values were in the low range of detection in our series (from 0 to 12, while the range of normalized intensity values for colon tissues was 0–31,330, 0–3,329 and 0–4,040 for experiments no. 1, 2 and 3, respectively), and this gene appeared to be slightly more expressed, in our samples, in the blood. Actually, in the last few years a very high number of genes claimed as "tissue specific" by classical analysis have proved to be expressed in many cell types if one uses RT-PCR techniques (capable of detecting a low level of expression) or cDNA microarray method (capable of scanning many tissues on a large-scale). In addition, *KRT20 *mRNA expression in colorectal cancers has been described as highly variable between 32 different tumours, with 3/32 cancers not expressing *KRT20 *mRNA, and *KRT20 *mRNA levels varying over three orders of magnitude [42,43]. This provides one explanation for the heterogeneity of *KRT20 *mRNA detection and quantification by various different authors, and for our failure to detect any significant amount of *KRT20 *mRNA in our samples by the cDNA microarray method.

Alternative approaches to get round the need for a clear cut-off between positive and negative samples could be quantitative RT-PCR or the use of a combination of several markers so as to attain statistical significance. However, it is often questioned whether a truly quantitative RT-PCR method can be consistent among many laboratories, as it is commonly difficult to optimize and interpret [41,44,45]. Various different RT-PCR based methods have been widely used in the detection of specific tumour cell markers, with varying degrees of sensitivity and specificity. It has been demonstrated that, to avoid non-specific amplification and thus false positive results, the most important factor is the particular tumour marker selected, quite apart from the particular type of RT-PCR variant adopted [19]. Assays incorporating a combination of multiple markers seems a reasonable approach, although we think that the existence of a major locus functioning as a master marker could be a crucial feature for the applicability of the test [30]. From this point of view, our current results seem to have provided an indication, for the first time obtained through genome-scale screening, that a suitable and consistent mRNA marker for detection of epithelial cells in the blood may actually not be present in the cytoplasm of colon-type cells, suggesting that new approaches to this problem need to be devised in the future. However, considering that our analysis investigated 20,000 out of about 25,000 protein-coding human genes, and that many genes could be expressed in tissue-specific splicing isoforms for which microarray platforms are not yet available, there is also the possibility that new microarray platforms with adjunctive probes for mRNAs or for specific isoform-splicing mRNAs, as well as platforms with probes for microRNAs [46], will reveal an RNA marker suitable for reliably detecting epithelial cells in the human blood.

## Competing interests

The author(s) declare that they have no competing interests.

## Authors' contributions

All authors were involved in the research presented and drafted and approved the final manuscript.

## Pre-publication history

The pre-publication history for this paper can be accessed here:



## Figures and Tables

**Table 1:** Colon cancer patients and control subject

Patient no.	Age	Sex	Stage^a^	Therapy
1	62	F	T3N1M0	S.R.
2	73	F	T4NXM1	B.P.
Controls				
3	83	M		
4	83	M		
5	84	F		
6	79	M		

^a^Staging according to American Joint Committee on Cancer (AJCC) and International Union Against Cancer. M, male; F, female. B.P., by-pass; S.R., sigmoid resection.

**Table 2:** Primers used for RT-PCR

**Primer**	**Sequence (5' → 3')**	**Direction**	**Nt 5'^a^**	**Expected size (bp)**
*PRSS22*	ctatcatcgactcggaagtc	f	668	676
	gatatgtgggcagggttaca	r	1343	
				
*RANBP3*	gctcgtacgagtgtagacactgggc	f	2195	191
	agggacctcgacagagctgtgcct	r	2385	
				
*TMEM69*	gatgtctctccagcaaaacttttc	f	281	299
	ctggctccataagccatctgag	r	579	
				
*B2M*	gcgggcattcctgaagctgac	f	25	586
	tacatcaaacatggagacagcac	r	610	

^a ^Nt 5' indicates the position of the nucleotide (Nt) at the 5' end of the primer. [GenBank:AB021288 (*B2M*), AB010779 (*PRSS22*), NM_003624 (*RANBP3*), NM_016486 (*TMEM69*)]. B2M, β-2 microglobulin; f, forward; r, reverse; bp, base pair.

**Table 3:** Gene data summary.

**Gene symbol**	**Gene description**	**Function**	Summary*
*PRSS22*	Protease, serine, 22 (Location: 16p13.3)	Serine-type endopeptidase activity	Member of the trypsin family of serine proteases. Enzyme expressed in the airways in a developmentally regulated manner. Serine proteases are involved in blood coagulation, growth factor processing, the digestion of nutrients, and cell migration. Northern hybridization showed that prosemin is expressed predominantly in the pancreas and weakly in the prostate and cerebellum.
*RANBP3*	RAN binding protein 3 (Location: 19p13.3)	Ran GTPase binding	The RAN GTPase plays an essential role in the transport of macromolecules between the cytoplasm and the nucleus, cell cycle progression, spindle assembly, nuclear organization and nuclear envelope (NE) assembly. RANBP3 (RAN binding protein 3) contains FXFG motifs characteristic of a subgroup of nucleoporins, and a C-terminal domain showing similarity to RANBP1.
*TMEM69*	Transmembrane protein 69 (Location: 17q21.2)	Unknown	-

*

## References

[B1] Etzioni R, Urban N, Ramsey S, McIntosh M, Schwartz S, Reid B, Radich J, Anderson G, Hartwell L (2003). The case for early detection. Nat Rev Cancer.

[B2] Greenlee RT, Hill-Harmon MB, Murray T, Thun M (2001). Cancer statistics, 2001. CA Cancer J Clin.

[B3] Nicholson FB, Barro JL, Atkin W, Lilford R, Patnick J, Williams CB, Pignone M, Steele R, Kamm MA (2005). Population screening for colorectal cancer. Aliment Pharmacol Ther.

[B4] Muller HM, Oberwalder M, Fiegl H, Morandell M, Goebel G, Zitt M, Muhlthaler M, Ofner DM, Margreiter R, Widschwendter M (2004). Methylation changes in faecal DNA: a marker for colorectal cancer screening?. The Lancet.

[B5] Weidner N, Carroll PR, Flax J, Blumenfeld W, Folkman J (1993). Tumor angiogenesis correlates with metastasis in invasive prostate carcinoma. Am J Pathol.

[B6] Martin KJ, Graner E, Li Y, Price LM, Kritzman BM, Fournier M, Rhei E, Pardee AB (2001). High-sensitivity array analysis of gene expression for the early detection of disseminated breast tumor cells in peripheral blood. Proc Natl Acad Sci USA.

[B7] Ross AA, Cooper BW, Lazarus HM, Mackay W, Moss TJ, Ciobanu N, Tallman MS, Kennedy MJ, Davidson NE, Sweet D, Winter C, Akard L, Jansen J, Copelan E, Meagher RC, Herzing RH, Klumpp TR, Kahn DG, Warner NE (1993). Detection and viability of tumor cells in peripheral blood stem cell collections from breast cancer patients using immunocytochemical and clonogenic assay techniques. Blood.

[B8] Smerage JB, Hayes DF (2006). The measurement and therapeutic implications of circulating tumour cells in breast cancer. Brit J Cancer.

[B9] Cristofanilli M, Budd GT, Ellis MJ, Stopeck A, Matera J, Craig Miller M, Reuben JM, Doyle GV, Allard WJ, Terstappen L, Hayes FD (2004). Circulating Tumor Cells, Disease Progression, and Survival in Metastatic Breast Cancer. New Eng J Med.

[B10] Burchill SA, Bradbury MF, Pittman K, Southgate J, Smith B, Selby P (1995). Detection of epithelial cancer cells in peripheral blood by reverse transcriptase-polymerase chain reaction. Br J Cancer.

[B11] Ohlmann CH, Ozgur E, Schrader AJ, Konrad L, Hofmann R, Engelmann U, Heidenreich A (2006). Detection of circulating tumor cells in patients with renal cell carcinoma by reverse transcriptase polymerase chain reaction for G250/MNCA-9: results of a prospective trial. Urol Oncol.

[B12] Douard R, Wind P, Sales JP, Landi B, Berger A, Benichou J, Gayral F, Loric S, Cugnenc PH (2006). Long-term prognostic value of detection of circulating colorectal cancer cells using CGM2 reverse transcriptase-polymerase chain reaction assay. Surgery.

[B13] Benoy IH, Elst H, Philips M, Wuyts H, Van Dam P, Scharpe S, Van Marck E, Vermeulen PB, Dirix LY (2006). Real-time RT-PCR detection of disseminated tumour cells in bone marrow has superior prognostic significance in comparison with circulating tumour cells in patients with breast cancer. Br J Cancer.

[B14] Maas RA, Bruning PF, Breedijk AJ, Top B, Peterse HL (1995). Immunomagnetic purification of human breast carcinoma cells allows tumor-specific detection of multidrug resistance gene 1-mRNA by reverse transcriptase polymerase chain reaction in fine-needle aspirates. Lab Invest.

[B15] Molnar B, Ladanyi A, Tanko L, Sreter L, Tulassay Z (2001). Circulating tumor cell clusters in the peripheral blood of colorectal cancer patients. Clin Cancer Res.

[B16] Weihrauch MR, Skibowski E, Draube A, Geller A, Tesch H, Diehl V, Bohlen H (2002). Immunomagnetic enrichment and detection of isolated tumor cells in bone marrow of patients with epithelial malignancies. J Clin Oncol.

[B17] Ree AH, Engebraaten O, Hovig E, Fodstad O (2002). Differential display analysis of breast carcinoma cells enriched by immunomagnetic target cell selection: gene expression profiles in bone marrow target cells. Int J Cancer.

[B18] Raynor M, Stephenson SA, Walsh DC, Pittman KB, Dobrovic A (2002). Optimisation of the RT-PCR detection of immunomagnetically enriched carcinoma cells. BMC Cancer.

[B19] Gilbey AM, Burnett D, Coleman RE, Holen I (2004). The detection of circulating breast cancer cells in blood. J Clin Pathol.

[B20] Guo J, Xiao B, Zhang X, Jin Z, Chen J, Qin L, Mao X, Shen G, Chen H, Liu Z (2004). Combined use of positive and negative immunomagnetic isolation followed by real-time RT-PCR for detection of the circulating tumor cells in patients with colorectal cancers. J Mol Med.

[B21] Smirnov DA, Zweitzig DR, Foulk BW, Miller MC, Doyle GV, Pienta KJ, Meropol NJ, Weiner LM, Cohen SJ, Moreno JG, Connelly MC, Terstappen LW, O'Hara SM (2005). Global gene expression profiling of circulating tumor cells. Cancer Res.

[B22] Lloyd JM, McIver CM, Stephenson SA, Hewett PJ, Rieger N, Hardingham JE (2006). Identification of early-stage colorectal cancer patients at risk of relapse post-resection by immunobead reverse transcription-PCR analysis of peritoneal lavage fluid for malignant cells. Clin Cancer Res.

[B23] Fournier MV, Guimaraes da Costa F, Paschoal ME, Ronco LV, Carvalho MG, Pardee AB (1999). Identification of a gene encoding a human oxysterol-binding protein-homologue: a potential general molecular marker for blood dissemination of solid tumors. Cancer Res.

[B24] Aquino A, Prete SP, Balduzzi A, Formica V, Fossile E, Bonmassar L, Concolino F, Bonmassar E, Graziani G (2002). Treatment of peripheral blood with staurosporine increases detection of circulating carcinoembryonic antigen positive tumor cells. Int J Cancer.

[B25] Clarke LE, Leitzel K, Smith J, Ali SM, Lipton A (2003). Epidermal growth factor receptor mRNA in peripheral blood of patients with pancreatic, lung, and colon carcinomas detected by RT-PCR. Int J Oncol.

[B26] Cillo U, Navaglia F, Vitale A, Molari A, Basso D, Bassanello M, Brolese A, Zanus G, Montin U, D'Amico F, Ciarleglio FA, Carraro A, Bridda A, Burra P, Carraro P, Plebani M, D'Amico DF (2004). Clinical significance of alpha-fetoprotein mRNA in blood of patients with hepatocellular carcinoma. Clin Chim Acta.

[B27] Dandachi N, Balic M, Stanzer S, Halm M, Resel M, Hinterleitner TA, Samonigg H, Bauernhofer T (2005). Critical evaluation of real-time reverse transcriptase-polymerase chain reaction for the quantitative detection of cytokeratin 20 mRNA in colorectal cancer patients. J Mol Diagn.

[B28] Giribaldi G, Procida S, Ulliers D, Mannu F, Volpatto R, Mandili G, Fanchini L, Bertetto O, Fronda G, Simula L, Rimini E, Cherchi G, Bonello L, Maule MM, Turrini F (2006). Specific detection of cytokeratin 20-positive cells in blood of colorectal and breast cancer patients by a high sensitivity real-time reverse transcriptase-polymerase chain reaction method. J Mol Diagn.

[B29] Zieglschmid V, Hollmann C, Bocher O (2005). Detection of disseminated tumor cells in peripheral blood. Crit Rev Clin Lab Sci.

[B30] Solmi R, De Sanctis P, Zucchini C, Ugolini G, Rosati G, Del Governatore M, Coppola D, Yeatman TJ, Lenzi L, Caira A, Zanotti S, Taffurelli M, Carinci P, Valvassori L, Strippoli P (2004). Search for epithelial-specific mRNAs in peripheral blood of patients with colon cancer by RT-PCR. Int J Oncol.

[B31] Engels WR (1993). Contributing software to the internet: the Amplify program. Trends Biochem Sci.

[B32] Davis LG, Kuehl WM, Battey JF (1994). Basic methods in molecular Biology.

[B33] http://apollo11.isto.unibo.it/suppl/solmi2006/.

[B34] Quackenbush J (2002). Microarray data normalization and transformation. Nat Genet.

[B35] Racila E, Euhus D, Weiss AJ, Rao C, McConnell J, Terstappen LW, Uhr JW (1998). Detection and characterization of carcinoma cells in the blood. Proc Natl Acad Sci USA.

[B36] Ashworth TR (1869). A case of cancer in which cells similar to those in the tumours were seen in the blood after death. Aus Med J.

[B37] Kramer BS, Gohagan JK, Prorok PC (1999). Cancer screening: theory and practice. Marcel Dekker New York.

[B38] Pinheiro NA, Caballero OL, Soares F, Reis LF, Simpson AJ (2001). Significant overexpression of oligophrenin-1 in colorectal tumors detected by cDNA microarray analysis. Cancer Lett.

[B39] Agrawal D, Chen T, Irby R, Quackenbush J, Chambers AF, Szabo M, Cantor A, Coppola D, Yeatman TJ (2002). Osteopontin identified as lead marker of colon cancer progression, using pooled sample expression profiling. J Natl Cancer Inst.

[B40] Mitsui S, Okui A, Kominami K, Konishi E, Uemura H, Yamaguchi N (2005). A novel serine protease highly expressed in the pancreas is expressed in various kinds of cancer cells. FEBS J.

[B41] Bustin SA, Mueller R (2006). Real-time reverse transcription PCR and the detection of occult disease in colorectal cancer. Mol Aspects Med.

[B42] Lassmann S, Bauer M, Soong R, Schreglmann J, Tabiti K, Nahrig J, Ruger R, Hofler H, Werner M (2002). Quantification of CK20 gene and protein expression in colorectal cancer by RT-PCR and immunohistochemistry reveals inter- and intratumour heterogeneity. J Pathol.

[B43] Schuster R, Max N, Mann B, Heufelder K, Thilo F, Grone J, Rokos F, Buhr HJ, Thiel E, Keilholz U (2004). Quantitative real-time RT-PCR for detection of disseminated tumor cells in peripheral blood of patients with colorectal cancer using different mRNA markers. Int J Cancer.

[B44] Freeman WM, Walker SJ, Vrana KE (1999). Quantitative RT-PCR: pitfalls and potential. Biotechniques.

[B45] Halford WP (1999). The essential prerequisites for quantitative RT-PCR. Nat Biotechnol.

[B46] Esquela-Kerscher A, Slack FJ (2006). Oncomirs – microRNAs with a role in cancer. Nat Rev Cancer.

